# HGFK1 Enhances the Anti-Tumor Effects of Angiogenesis Inhibitors via Inhibition of CD90+ CSCs in Hepatocellular Carcinoma

**DOI:** 10.3390/ph17050645

**Published:** 2024-05-16

**Authors:** Tao Li, Ling Liu, Li Li, Xiaoxuan Yao, Xiaoyuan Hu, Jiaxing Cheng, Zhenpu Chen, Jiyin Guo, Ruilei Li, Chunlei Ge, Marie Chia-Mi Lin, Hong Yao

**Affiliations:** 1 Cancer Biotherapy Center & Cancer Research Institute, Peking University Cancer Hospital Yunnan, Yunnan Cancer Hospital, The Third Affiliated Hospital of Kunming Medical University, Kunming 650106, China; litaove@163.com (T.L.);; 2 Cancer Institute, Xuzhou Medical University, Xuzhou 221002, China

**Keywords:** hepatocellular carcinoma (HCC), HGFK1, CD90+ cancer stem cells (CSCs), chemotherapy drug cisplatin (CDDP), nanoparticles

## Abstract

The combination of anti-angiogenesis agents with immune-checkpoint inhibitors is a promising treatment for patients with advanced hepatocellular carcinoma (HCC); however, therapeutic resistance caused by cancer stem cells present in tumor microenvironments remains to be overcome. In this study, we report for the first time that the Kringle 1 domain of human hepatocyte growth-factor α chain (HGFK1), a previously described anti-angiogenesis peptide, repressed the sub-population of CD90+ cancer stem cells (CSCs) and promoted their differentiation and chemotherapy sensitivity mainly through downregulation of pre-Met protein expression and inhibition of Wnt/β-catenin and Notch pathways. Furthermore, we showed that the i.p. injection of PH1 (a tumor-targeted and biodegradable co-polymer), medicated plasmids encoding Endostatin (pEndo), HGFK1 genes (pEndo), and a combination of 50% pEndo + 50% pHGFK1 all significantly suppressed tumor growth and prolonged the survival of the HCC-bearing mice. Importantly, the combined treatment produced a potent synergistic effect, with 25% of the mice showing the complete clearance of the tumor via a reduction in the microvessel density (MVD) and the number of CD90+ CSCs in the tumor tissues. These results suggest for the first time that HGFK1 inhibits the CSCs of HCC. Furthermore, the combination of two broad-spectrum anti-angiogenic factors, Endo and HGFK1, is the optimal strategy for the development of effective anti-HCC drugs.

## 1. Introduction

Tumors are frequently composed of heterogeneous cell types, in which a rare population named cancer stem cells (CSCs) or tumor-initiating cells drives tumor initiation and growth [1]. CSCs share certain properties with normal stem or progenitor cells, such as self-renewal, indefinite systemic cell division, and proliferation. Furthermore, CSCs also give rise to tumor metastasis, recurrence, and chemotherapy/radiotherapy resistance [2]. Therefore, the development of therapeutic strategies that effectively target CSCs could have a major impact on cancer-patient survival.

The vascular niche is an ideal microenvironment for the growth and maintenance of adult stem cells in normal organs. This observation also extends to tumors, in which CSCs are often present near tumor blood vessels, and the vascular niche directly promotes the growth of CSCs via juxtracrine and paracrine effects. The crosstalk between CSCs and vascular genesis raises the possibility that the most effective anti-angiogenic therapy should target both endothelial and perivascular cell lineages, including CSCs. Hepatocellular carcinoma (HCC) is one of the most vascular solid tumors, the carcinogenesis of which angiogenesis plays an important role. Several independent sub-populations of cells have shown CSC properties in HCC, including CD133+, CD90+, and epithelial cell-adhesion molecule (EpCAM)+ cells, or the selected side population (SP) cells in Hoechst dye-staining [3]. Among them, CD90+ HCC cells have been recognized as a CSC population with the properties of vascular endothelial cells and a high incidence of distant organ metastasis.

Human hepatocyte growth factor (HGF) is a scatter protein previously known to induce the proliferation, migration, angiogenesis, and survival of cancer cells. Recently, HGF has been shown to be present in the tumor microenvironment and plays a critical role in the maintenance of the stemness properties of CSCs, suggesting that molecules that inhibit the HGF pathway may be potential anti-CSC reagents [4,5]. The kringle 1 domain of HGFα-chain (HGFK1) is the core domain of the first kringle (K1) and the N-terminal heparin-binding domain, which acts as a receptor antagonist of HGF in the absence of heparin, which is considered to be an effective anti-angiogenic factor [6]. Previously, Lin et al. [7] showed that the recombinant polypeptide of HGFK1 (rHGFK1) inhibited the basic fibroblast growth factor (bFGF)-induced proliferation of bovine aortic endothelial cells and suppressed tumor growth and metastasis by inhibiting tumor neovascularization in an established rat orthotopic hepatocellular carcinoma (HCC) model. Our recent studies have further demonstrated that HGFK1 exerts anti-tumoral and radio-sensitizing effects via the inhibition of mesenchymal-to-epithelial transition factor (Met) in glioblastoma [8], and it can also enhance the anti-tumor effect of sorafenib by inhibiting drug-induced autophagy and stemness in renal cell carcinoma [9]. Transarterial embolization (TAE) combined with HGFK1 showed a significant anti-tumor effect on hepatocellular carcinoma [10]. However, the functions of HGFK1 in the CSCs of HCC remain to be defined.

In this study, we investigated the effects of HGFK1 and Endostatin, the commonly used endogenous anti-angiogenesis molecule, on the CD90+ CSCs of HCC both in vitro and in vivo. Specifically, we produced a polymeric nanoparticle, which was formed with a previously developed cationic co-polymer, the mixture consisting of PEI600-CyD-FA and PEG-PEI600-CyD (PH1) and CpG-free plasmids encoding the HGFK1 (pHGFK1) and Endostatin (pEndo) gene, respectively. We have shown that these polymeric nanoparticles could effectively deliver the therapeutic genes into the tumor of the liver in an orthotopic HCC mouse model via intraperitoneal (i.p.) injection. Furthermore, we have shown that the i.p. injection of PH1/pHGFK1, PH1/pEndostatin, and their nanoparticle-based combination treatment all significantly inhibited tumor growth via reducing the microvessel density (MVD) and the number of CD90+ CSCs of the tumor tissues in this model.

## 2. Results

### 2.1. The Difference between and Synergistic Effect of rHGFK1 and rEndo on the Proliferation of Endothelial Cells and Hepatocellular Carcinoma Cells

**Figure 1:**
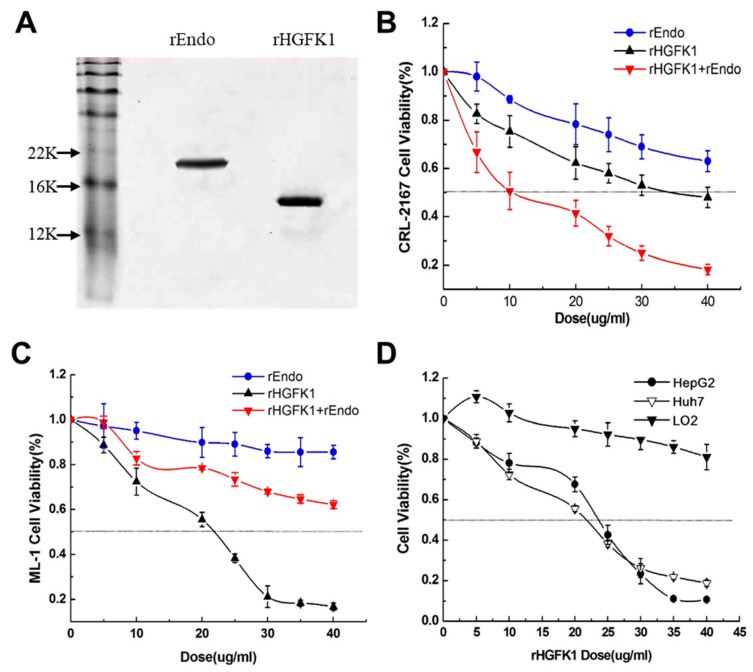
rHGFK1, rEndo, and their combination treatment inhibited the proliferation of endothelial cells and HCC cells. (**A**). Demonstration of the purification of rHGFK1 and rEndo proteins using a PAGE gel. (**B**,**C**). The effects of rHGFK1, rEndo, and their combination on the proliferation of endothelial cells (CRL-2167 cells) and hepatocellular carcinoma cells (ML-1 cells) were detected via MTT assays. (**D**). MTT assays show the proliferation-inhibiting effect of rHGFK1 on the hepatocellular carcinoma cell line HepG2, Huh7, and the human hepatic cell line LO2. Each bar represents the mean of three independent measurements (±SEM).

To evaluate the functions of two previously defined anti-angiogenetic molecules, we first expressed and purified recombinant polypeptides HGFK1 (rHGFK1) and Endostatin (rEndo) (Figure 1A) and, then, studied their effects on the proliferation of endothelial cells and HCC cells via the methylthiazolyldiphenyl-tetrazolium bromide (MTT) assay. As shown in Figure 1B, rHGFK1 and rEndo, either alone or in combination, dose-dependently inhibited the percentage viability (PV) of CRL-2167 cells, i.e., mouse endothelial cells. The combination of rHGFK1 (50%) and rEndo (50%) (rHGFK1+rEndo) showed a strong synergistic effect (with an IC_50_ of approximately 10 µg/mL as compared to 40 μg/mL for rHGFK1 and >40 µg/mL for rEndo), suggesting that the anti-angiogenetic efficacy of rHGFK1 was better than that of rEndo, and the combination of rHGFK1 and rEndo could synergistically enhance their potencies in inhibiting angiogenesis. However, for ML-1 cells, a mouse HCC cell line, only rHGFK1 treatment was effective in inhibiting cell proliferation (Figure 1C), with an IC_50_ around 50 μg/mL. The rEndo produced no significant effect at concentrations lower than 50 µg/mL, and the combination of rHGFK1 and rEndo only produced 50% of the effect (Figure 1C). For HepG2 and Huh7 cells, two human HCC cell lines, rHGFK1 treatment also significantly suppressed their proliferation, but for LO2 cells, a human hepatic cell line, it had no significant effect, even at a dose of over 30 µg/mL (Figure 1D). These results suggest that, although HGFK1 and Endo showed excellent effects in inhibiting the proliferation of endothelial cells alone or in cooperation, it was HGFK1 rather than Endo effectively inhibiting the proliferation of HCC cells, and HGFK1 had no inhibitory effect on normal hepatic cell lines.

### 2.2. rHGFK1 Inhibits the Stemness and Self-Renewal of CD90+ CSCs of HCC Cells In Vitro

**Figure 2:**
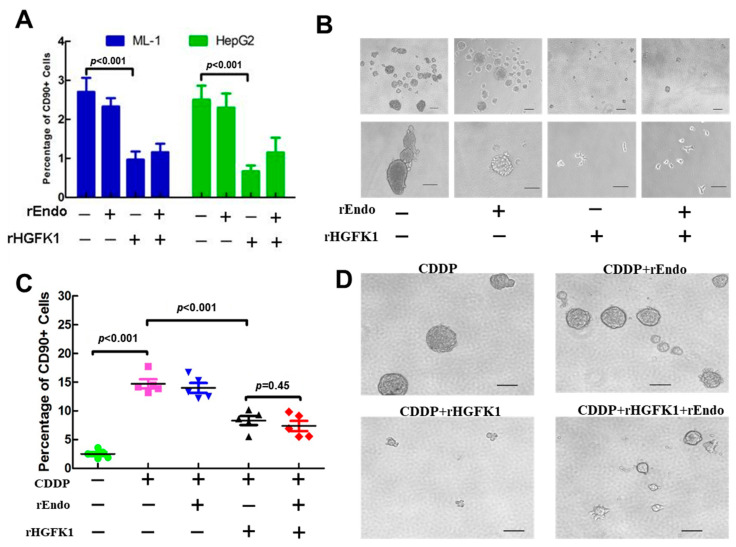
rHGFK1 inhibited the stemness and self-renewal of CD90+ CSCs of HCC cells and enhanced the chemotherapy sensitivity of CDDP-enriched HepG2 cells. (**A**). The effects of rEndo (10 µg/mL), rHGFK1 (10 µg/mL), and rEndo (5 µg/mL) + rHGFK1 (5 µg/mL) on the percentage of CD90+ sub-populations, determined via flow-cytometry analysis, in two HCC cell lines treated for 4 days. (**B**). Phase-contrast microscopy determined the spherical formation ability to evaluate the self-renewal ability of CSCs in vitro; scale bars, 100 μm. CD90+ CSC sub-populations were isolated from Huh7 cells via flow cytometry and treated with 10 µg/mL rEndo, 10 µg/mL rHGFK1, and 5 µg/mL rEndo+5 µg/mL rHGFK1. (Under the condition of 10 µg/mL, the difference in the spherical formation ability of CD90+ CSCs was more obvious.) (**C**,**D**). The spherical formation ability of Huh7 CD90+CSC sub-populations treated with 5 µg/mL CDDP combined with 10 µg/mL rEndo, 10 µg/mL rHGFK1, and 5 µg/mL rEndo +5 µg/mL rHGFK1; data are shown as mean  ±  SEM; scale bars, 100 μm. Each bar represents the mean of three independent measurements (±SEM). *p*-values for the comparison of each group were determined via one-way ANOVAs.

Previous studies have shown that CD90+ CSCs of HCC cells have the characteristics of tumorigenicity, strong invasiveness, and metastasis [11], so their cell number and proliferation ability are often regarded as indicators of the stemness of CSCs. To this end, we evaluated the effects of rEndo, rHGFK1, and rEndo+rHGFK1 on the percentage of CD90+ sub-populations via a flow-cytometry analysis of two HCC cell lines. As shown in Figure 2A, only the rHGFK1 and rEndo+rHGFK1 treatments significantly decreased the percentage of CD90+ sub-populations in ML-1 cells and HepG2 cells (reduced to 35% and 42%, and 26% and 46%, of that of the control value, respectively), indicating that rHGFK1 had a significant inhibitory effect on the self-renewal of CD90+ CSCs of HCC cells, while Endo did not.

Furthermore, we measured the spherical formation ability of CSCs to evaluate their self-renewal ability in vitro. We isolated the CD90+ sub-population of CSCs from Huh7 cells by flow-cytometry sorting, and treated cells with rEndo, rHGFK1, or rEndo+rHGFK1 for 4 days; and determined their spherical formation ability through phase-contrast microscopy. As shown in Figure 2B, only the rHGFK1 and rHGFK1+rEndo treatments significantly reduced the volume of the hepatospheres compared to that observed with the control PBS and rEndo treatments, in which some hepatospheres were spherically grown, removed, and adhered on the bottom of the ultra-low attached plate. These results suggest that rHGFK1 inhibited the spherical formation ability and promoted the differentiation of CD90+ CSCs. Importantly, the combination of rHGFK1 (50%) and rEndo (50%) also showed synergistic effects.

### 2.3. rHGFK1 Enhanced the Chemotherapy Sensitivity of CDDP-Enriched HepG2 Cells

CSCs show chemotherapy resistance due to their abilities to induce quiescence, the expression of ABC drug pumps, the high expression of antiapoptotic proteins, and resistance to DNA damage [12,13]. A low dose of the chemotherapy drug cisplatin (CDDP) has been used to enrich CSCs in HCC [14]. In our study, we further combined with the chemotherapy drug cisplatin (CDDP) to explore the effect of rHGFK1 on the self-renewal ability of CSCs. The result showed that CDDP treatment increased the mean percentage of CD90+ cells to 5.8-fold that of the control PBS treatment among HepG2 cells (Figure 2C), and the chemosensitivity of HCC stem cells decreased, causing the cells to be enriched. Strikingly, when rHGFK1 or rHGFK1+rEndo was added to the CDDP-enriched medium, the mean percentage of CD90+ CSCs was reduced to 0.56-fold (56%) or 0.5-fold (50%) that of the CDDP-enriched cells. In contrast, in the CDDP+rEndo (10 µg/mL)-treated cells, the mean percentage of CD90+ cells did not change significantly (Figure 2C), suggesting that rHGFK1 and the rHGFK1+ rEndo could synergistically enhance the chemotherapy sensitivity and inhibit the self-renewal ability of the CD90+ CSCs of HCC cells.

These effects were further confirmed via a spherical formation assay with CD90 antibody-sorted CSCs. As shown in Figure 2D, phase-contrast microscopy showed that the rHGFK1+CDDP treatment significantly reduced the volume of the hepatospheres but did not change the morphology compared to the CDDP-treated control. The volume of the hepatospheres decreased in the RHGFK1+rEndo+CDDP treatment group, but some of them attached to ultra-low-adherent panels, suggesting that rHGFK1 and rHGFK1+rEndo treatment inhibited the spherical formation of CSCs and promoted the differentiation of CSCs. In summary, rHGFK1, especially when used in combination with CDDP, can inhibit the spheroid formation ability of CD90+ CSC cells, and may promote their differentiation in the presence of other chemotherapeutic agents and enhance the sensitivity of HCC cells to chemotherapeutic agents.

### 2.4. rHGFK1 Inhibited CSCs via Decreasing the Activity Levels of Pre-Met, Wnt/β-Catenin, and Notch Pathways

**Figure 3:**
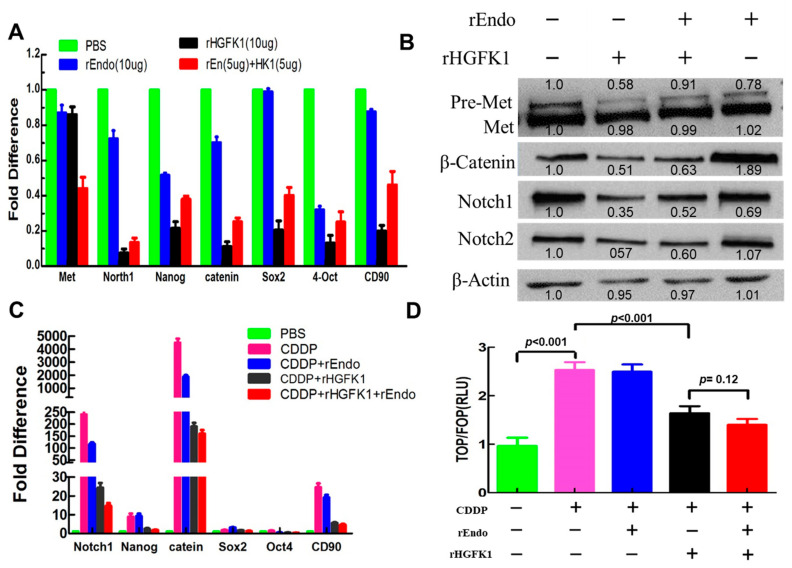
The expression levels of genes and proteins in the HGF/Met, Wnt/β-Catenin, and Notch pathways. (**A**,**B**). The gene (**A**) and protein (**B**) expression levels in rHGFK1 (10 µg/mL)-treated, rEndo (5 µg/mL)-treated, and rHGFK1 (5 µg/mL) + rEndo (5 µg/mL)-treated HepG2 cells determined via qPCR. (**C**). The gene expression level was determined via qPCR in HepG2 cells treated with 5 µg/mL CDDP combined with 10 µg/mL rEndo, 10 µg/mL rHGFK1, and 5 µg/mL rEndo + 5 µg/mL rHGFK1. (**D**). The TOP/FOP reporter system was used to evaluate the activity of the Wnt/β-catenin pathway. The signal value of TOP/FOP (RLU) is not only a response to the transcriptional activity of target TCF/LEF, but also indirectly reflects the activation of the Wnt pathway, which shows a positive correlation. Each bar represents the mean of three independent measurements (±SEM). *p*-values for comparison of each group were determined via one-way ANOVAs.

The hepatocyte growth factor (HGF) ligand binds to the receptor tyrosine kinase (Met), which can transmit signals from the extracellular matrix to the cytoplasm and regulate many physiological processes, including proliferation, scattering, morphogenesis, and survival [15]. Therefore, we also determined the effects of rHGFK1 on the HGF/C-Met pathway. However, interestingly, in rHGFK1-treated HepG2 cells, the gene expression level of Met was not significantly reduced compared to that of the control (Figure 3A), but the protein level of pre-Met was significantly lower than that of the control (Figure 3B). These results suggest that the reduction in pre-Met protein in rHGFK1-treated cells should be mainly attributed to the protein degradation but not to the downregulation of gene expression. However, the mechanisms of pre-Met degradation induced by rHGFK1 remain to be elucidated in the future.

The conserved Wnt/β-Catenin and Notch pathways regulate the pluripotency of stem cells and determine the differentiation fate of cells during development [16]. In order to understand whether the molecular mechanism of HGFK1 inhibiting the stemness of CSCs in HCC is associated with the Wnt/β-catenin and Notch signal pathways, we further determined the mRNA and protein levels of associated genes, including β-catenin, Nnotch1, and stemness-associated genes, including Nanog, Sox-2, 4-Oct, and CD90. As shown in Figure 3A, in rHGFK1-treated and rHGFK1+rEndo-treated HepG2 cells, the mRNA levels of β-catenin and Notch1 were reduced to approximately 0.13- and 0.19-fold and 0.07- and 0.15-fold of those in the control group, and the mRNA levels of the stemness-related genes Nanog, Sox-2, 4-Oct, and CD90 also showed a similar downward trend. Correspondingly, in these treatments, the protein-expression levels of non-phosphorylated β-catenin (np β-catenin), Notch1, and Notch2 were reduced to 0.4- and 0.2-fold, 0.47- and 0.70-fold, and 0.65- and 0.56-fold, respectively, of those in the control group (Figure 3B).

Consistently, similar results were also obtained in CDDP-enriched cells. As shown in Figure 3C, after 48 h of CDDP treatment, the gene expression of β-catenin (4400-fold), Notch1 (240-fold), and stemness-associated genes, including Nanog (8.9-fold) and CD90 (24-fold), was most dramatically upregulated, while the rHGFK1 and rHGFK1+rEndo treatments significantly inhibited the CDDP-induced upregulation of β-catenin, Notch1, and stemness-associated genes. These gene expression levels were, respectively, reduced to 12% and 11%, 35% and 30%, 26% and 20%, and 31% and 27% of those observed with CDDP treatment alone, indicating that HGFK1 could significantly inhibit the activation of the CDDP-induced Wnt/β-catenin and Notch signaling pathways, thus inhibiting the stemness and CDDP-enriched ability of CD90+ CSCs in HCC.

TOPFlash (reporter-gene plasmid) is a reporter-gene plasmid used to detect the level of β-catenin-mediated TCF/LEF transcriptional activity in the Wnt signaling pathway, in which LEF and TCF combine with Groucho to inhibit the expression of downstream genes regulated by Wnt. At the same time, the transcriptional complex formed by β-catenin and TCF/LEF in the nucleus can activate gene expression downstream of the Wnt signal pathway, so it can be used to detect the activation of this pathway. To this end, we further performed transcriptional reporter assays using the TOP/FOP reporter system to evaluate the activity of the Wnt/β-catenin pathway [17]. As shown in Figure 3D, the target-site TCF/LEF transcription activity of the CDDP-treated cells was 2.5-fold higher than that of the PBS-treated cells, while the TCF/LEF transcriptional activities of CDDP+rHGFK1- and CDDP+rHGFK1+rEndo-treated cells were decreased to 65% and 56% of that of the CDDP-treated cells, respectively. In contrast, the TCF transcription activity of CDDP+rEndo was not changed. These results suggest that CDDP treatment activated β-catenin/TCF transcription activity, which contributed to the enrichment of CDDP-induced CSCs, while rHGFK1 inhibited the activation, and rEndo synergistically enhanced the inhibition.

Therefore, the above experimental data suggest that both rHGFK1 and the combination of rHGFK1 and rEndo inhibited the activity of the Wnt/β-catenin and Notch signaling pathways, with the combination treatment exhibiting synergic effects.

### 2.5. Development of an Effective Liver- and Tumor-Targeting Gene Delivery Polymeric Nanoparticle System

**Figure 4:**
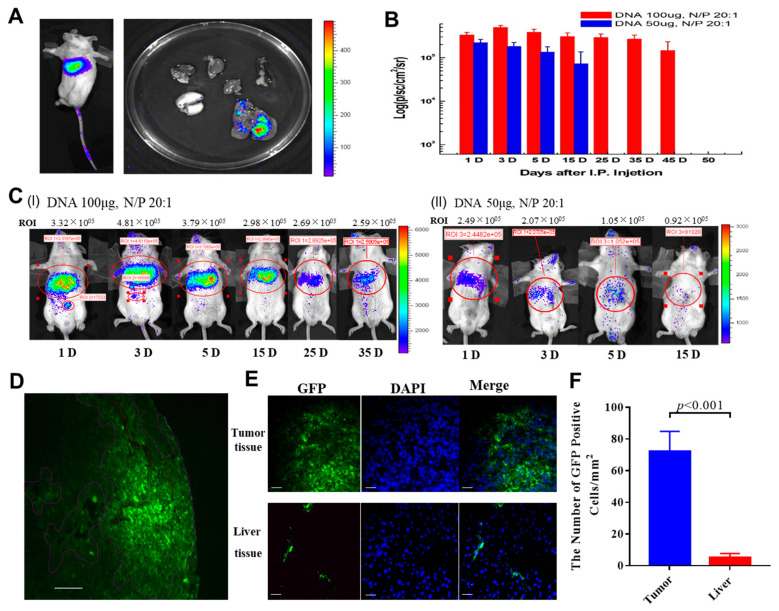
H1 and PH1 are effective liver- and tumor-targeting polymeric particles for drug delivery. (**A**). Distribution of H1/pORF-Luciferase (H1/pORF-Luc) nanoparticles injected into the tail vein in BALB/C mice. (**B**,**C**). The luciferase expression at different days after injection of 50 μg and 100 μg H1/pORF-Luc nanoparticles into mice (N/P 20:1). (**D**). Distribution and aggregation of PH1/pEGFP-positive cells in tumor tissues of mice after injection of PH1/pORF-EGFP nanoparticles at 48 h. Scale bars, 100 μm. (**E**,**F**). Immunohistochemical detection of the number of green-fluorescent protein-positive cells in tumor tissues and normal liver tissues. Each bar represents the mean of three independent measurements (±SEM). *p*-values for comparison of each group were determined via *t*-test analysis.

The excellent inhibitory effect of HGFK1 in the stemness and self-renewal of CD90+ CSCs makes it a potential therapeutic drug for HCC. However, an effective and long-lasting liver-targeting delivery system is necessary. Previously, we reported that, when administered via intertumoral injection, a PEI600-CyD-FA (H1)/plasmid polyplex nanoparticle was an effective, biodegradable, and low-toxicity gene delivery vector [18]. In this study, we found that H1/pORF-Luciferase (H1/pORF-Luc) nanoparticles accumulated in the upper abdomen of BALB/C mice after tail-vein injection; more specifically, they accumulated in the liver, but not in the lungs, heart, spleen, or kidneys (Figure 4A), indicating that the systemic administration of H1/pORF-DNA nanoparticles showed a trend of accumulating in the liver. Subsequently, the results for a CpG-free plasmid encoding the luciferase gene showed that H1/pORF-Luc nanoparticles produced sustained luciferase gene expression in the mouse upper abdomen (Figure 4B), with dynamic changes in total photon flux (TPF) that were time and dose dependent (Figure 4C). For the H1/pORF-Luc nanoparticles carrying 100 µg of DNA, the luciferase expression could be retained at above 50% until day 35 then declined to 25% on day 45, and it disappeared after day 15 with 50 μg of DNA. The dose-dependent phenomenon of gene expression suggested the presence of non-specific absorbance of H1/pORF-DNA nanoparticles passing through the Kupffer cells of the liver.

To facilitate more effective in vivo delivery, we developed a tumor-targeting polymeric nanoparticle vector system (named PH1) by conjugating the PEI-CyD backbone with polyethylene glycol (PEG, M.W. 3300). The system is a bifunctional polymer mixture of PEI600-CYD-FA (H1) and PEG-PEI600-CYD (P1) at the ratio of 1:1 with both passive and active tumor-targeting properties. To evaluate the tumor-targeting ability, PH1/pORF-EGFP nanoparticles were administered by intraperitoneal (i.p.) injection (N/P 20:1, DNA 100 µg) into the mice bearing orthotopic HCC. As shown in Figure 4D, at 48 h post-injection, the green-fluorescent protein (GFP)-positive cells were distributed and accumulated mainly inside the tumor tissues near the microvessels. Further stereological studies indicated that the number of GFP-positive cells was approximately 10 times higher in the tumor tissue than in the nearby normal liver tissue (Figure 4E,F). Taken together, these results suggest that PH1/pORF-EGFP nanoparticles were absorbed by a superior mesenteric vein into the interstitial via the leaking tumor microvessel and then internalized by the cancer cells, thus homing mainly to the tumor tissue of the liver. These results show that PH1 nanoparticles were an effective liver- and tumor-targeting gene delivery polymeric nanoparticle system.

### 2.6. PH1 Polymeric Nanoparticle-Mediated pORF-HGFK1, pORF-Endo, and Combined pORF-HGFK1+pORF-Endo Gene Therapies for the Treatment of HCC

**Figure 5:**
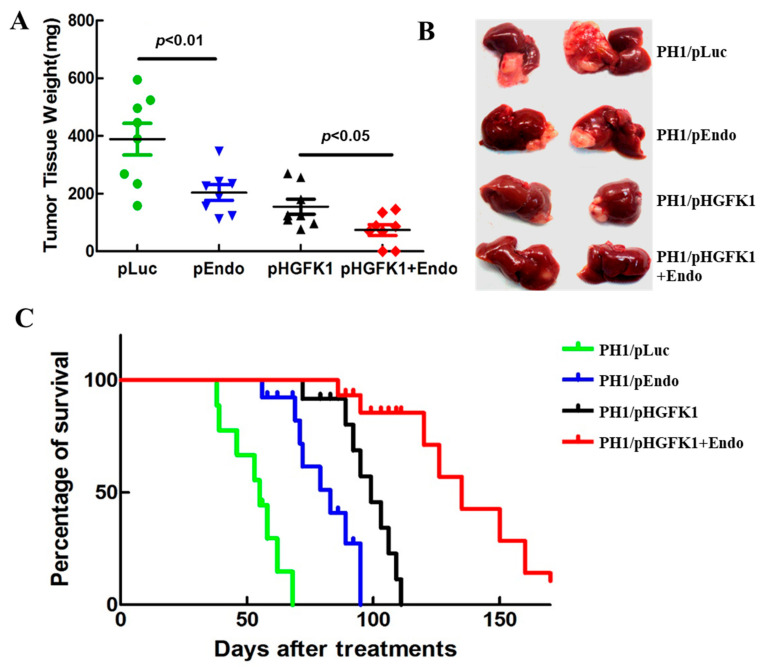
Anti-angiogenic effects of endostatin and HGFK1 on the treatment of the orthotopic HCC-bearing mice. (**A**). Tumor tissue weight after tail-vein injection of PH1/pORF-Luc, pORF-Endo, pORF-HGFK1, and pORF-Endo+ pORF-HGFK1 in orthotopic HCC-bearing mice. (**B**). The representative liver morphologies. On the 30th day after the first treatment, 6 mice in each group were sacrificed, and the liver tissues were taken and photographed. (**C**). The average survival time of orthotopic HCC-bearing mice treated with PH1 polymer nanoparticle-mediated pORF-HGFK1 and pORF-Endo gene therapies. Each bar represents the mean of three independent measurements (±SEM). The *p*-values for the comparison of each group were determined via one-way ANOVAs.

Based on the development of effective liver- and tumor-targeting gene delivery polymeric nanoparticle systems, we studied the efficacy of two anti-angiogenesis genes, Endostatin and HGFK1, on the treatment of the orthotopic HCC-bearing mice. Orthotopic HCC-bearing mice were established by injecting mouse HCC ML-1 cells into BALB/c mice, as described previously [19]. The mice were divided into four groups and treated via the i.p. injection of PH1/pORF-Luc, pORF-Endo, pORF-HGFK1, or pORF-Endo+ pORF-HGFK1. On day 30 after the first treatment, six mice from each group were sacrificed, the tumor mass was weighed, and the representative liver morphologies were recorded. The results showed that the average tumor weight in the pORF-Endo, pORF-HGFK1, and pORF-Endo+pORF-HGFK1 treatment groups decreased significantly, from 540 ± 81 (control was PH1/pORF-Luc treated) to 240 ± 65, 210 ± 50, and 85 ± 74 mg, respectively (Figure 5A). The combined pORF-Endo+pORF-HGFK1 treatment showed synergistic efficacy, with 25% of the mice completely cleared of any detectable tumor nodules (Figure 5B).

Furthermore, as shown in Figure 5C, the mean survival time increased significantly, from 52 days after the first treatment (control PH1/pORF-Luc treated) to 76, 97, and 134 days for the pORF-Endo, pORF-HGFK1, and pORF-Endo-pORF-HGFK1 treatment groups, respectively. Similarly, the combined pEndo+pHGFk1 treatment group exhibited the longest survival time, with 25% of the mice in this group living for more than 150 days. PH1 polymer nanoparticle-mediated pORF-HGFK1 and pORF-Endo gene therapies for HCC showed a beneficial effect.

### 2.7. pORF-HGFK1, pORF-Endo, and Combined pORF-Endo+pORF-HGFK1 Treatments Significantly Reduced the Tumor Microvessel Density and the Number of CD90-Positive Cells

**Figure 6:**
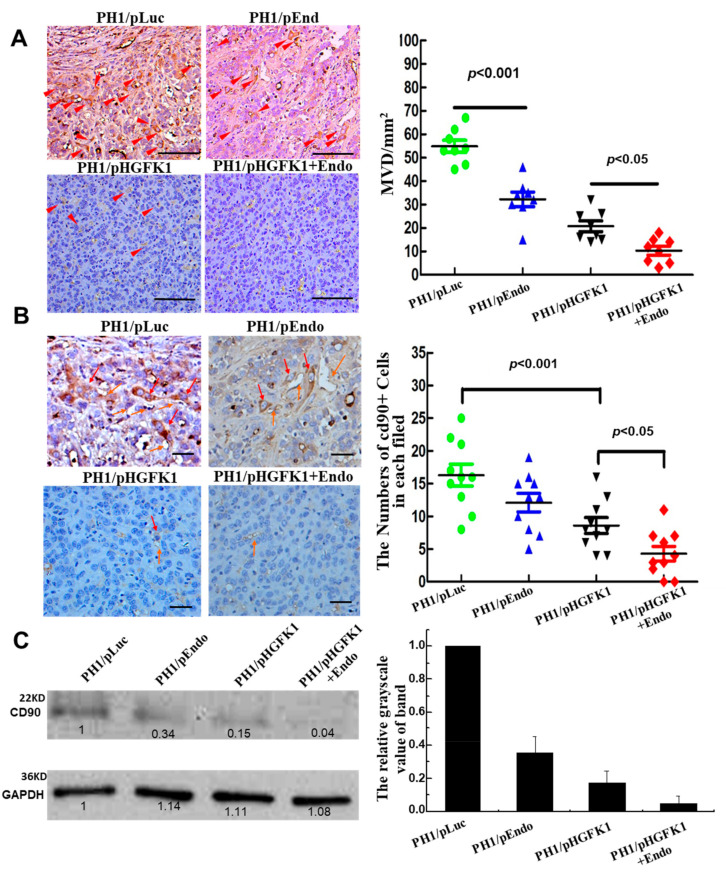
PH1 polymer nanoparticle-mediated pORF-HGFK1 and pORF-Endo gene therapies significantly reduced the tumor microvessel density and the number of CD90-positive cells. (**A**). The endothelial cell marker, CD31, was measured via immunostaining to determine the intra-tumor microvessel density (MVD); scale bars, 100 μm. The red arrow indicates the positive area. (**B**). The number of CD90-positive cells and the expression of CD90 protein in the tumor tissues were detected using immunohistochemistry to identify the pORF-HGFK1 and pORF-Endo gene therapy’s effects on CSCs; scale bars, 100 μm. The red arrow indicates the positive area. (**C**). The expression of the CD90 protein in the tumor tissues was detected via Western blotting experiments. The arrows in the picture represent different positive areas. Each bar represents the mean of three independent measurements (±SEM). *p*-values for the comparison of each group were determined via one-way ANOVAs.

On day 30 post-first treatment, the intra-tumor microvessel density (MVD) was determined via immunostaining for the endothelial cell marker CD31. As shown in Figure 6A, the MVD/mm^2^ of the tumor tissue in the mice treated with PH1/pORF-Endo (100 µg) and PH1/pORF-HGFK1 (100 µg) nanoparticles decreased to 50 and 33% of the control value, respectively. Importantly, the combined pORF-Endo+pORF-HGFK1 nanoparticle treatment produced a synergistic effect, as the MVD/mm^2^ was significantly reduced to only 8% of that of the control value.

To assess their effects on CSCs, we determined the number of CD90-positive cells and the level of CD90 protein expression in the tumor tissues via immunohistochemistry and Western blotting experiments. As shown in Figure 6B,C, a large number of CD90-positive cells were found in the tumor tissue from the control PH1/pORF-Luc-treated mice. The pORF-Endo and pORF-HGFK1 nanoparticle treatments reduced the number of CD90-positive cells to 50% and 40% of the control value, respectively. Again, a synergistic effect was observed in the combined pORF-Endo+pORF-HGFK1 treatment, with CD90-positive cells reduced to 9%. Moreover, the CD90-positive cells were frequently found around the microvessels of the tumor (red and orange arrows indicate the CD90-positive cells and microvessels, respectively), thereby indicating the possible close interactions and crosstalk between angiogenesis and CSCs. PH1/pORF-HGFK1 and PH1/pORF-Endo-HGFK1 may be effective at breaking the cross-linking relationship between angiogenesis and CSC, resulting in significant killing of CSCs and anti-tumor efficacy.

To further confirm these results, the expression level of the CD90 protein in the tumor tissue was determined through a Western blotting experiment, and the grayscale value of the band was quantified using the Bio-Red software package (QX Manager Software Standard Edition, Version 2.1). As shown in Figure 6C, pORF-Endo, pORF-HGFK1, and the combined pORF-Endo+pORF-HGFK1 nanoparticle treatment reduced the level of CD90 protein expression in the liver tumor tissues to 34%, 15%, and 4% of the value of the control pORF-Luc treatment group. Therefore, pORF-Endo, pORF-HGFK1, or the combined pORF-Endo+pORF-HGFK1 showed a significant effect in reducing tumor microvessel density and inhibiting stem cell proliferation.

### 2.8. Identification, Characterization, and Quantification of Differential Proteins in Different Treatment Groups after 2-DE Combined with MS

**Figure 7:**
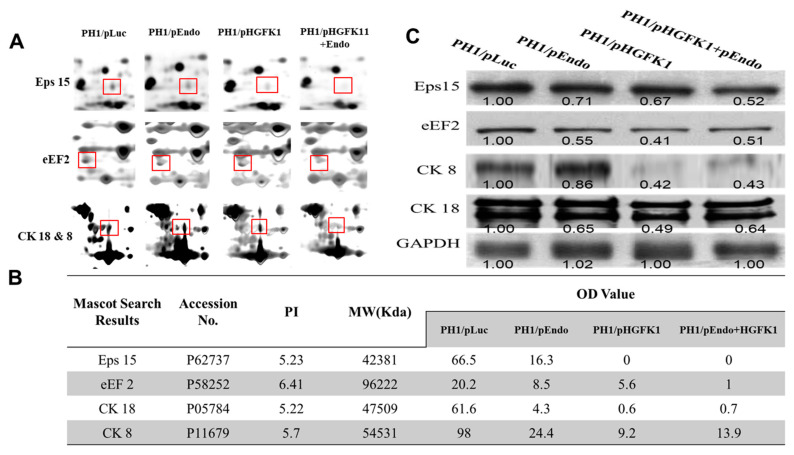
Identification, characterization, and quantification of differential proteins in liver tumor tissues treated with pORF-Luc (control), pORF-Endo, pORF-HGFK1, and the combined pORF-Endo+pORF-HGFK1 nanoparticles after 2-DE combined with MS analysis. (**A**). Meat proteins were separated by 2-DE (red box) and visualized with Sypro Ruby, and the gel image was subsequently scanned to obtain meat protein reference patterns and the total protein volume of spots. (**B**). The key protein information identified by 2-DE combined with MS, including PI (isoelectric point) and MW (molecular weight), and the OD value of each protein in liver tissues of different treatment groups. (**C**). Western blot to validate the expression of key proteins Eps15, eEF2, CK18, and CK8 in different treatment groups.

To investigate the molecular mechanisms, the protein-expression profile of the liver tumor tissues treated with pORF-Luc (control), pORF-Endo, pORF-HGFK1, and the combined pORF-Endo+pORF-HGFK1 nanoparticle was studied following proteomic approaches and using two-dimensional electrophoresis (2-DE) coupled to mass spectrometry (MS) analysis and Western blot validation (Figure 7). A total of 43 proteins were found to be dysregulated. Among them, the expression levels of 23 proteins declined in all three treatment groups compared to the control group. According to their magnitudes of change and their predicted biological functions, we are particularly interested in four proteins, i.e., epidermal growth-factor receptor pathway substrate 15 (Eps15), elongation factor 2 (eEF2), and cytokeratin polypeptides 8 and 18 (CK8/CK18). As compared with the control, the expression of these four proteins significantly decreased in the tumor tissue of the mice that had received all three treatments (Figure 7A,B). Among them, pORF-HGFK1 exhibited significantly more potent effects on Eps15, CK18, and CK8 compared to pEndo, suggesting that these three proteins may have contributed to the anti-cancer stem-cell activity of HGFK1 (Figure 7B). We also observed a marked synergistic effect of the combined pORF-Endo+pORF-HGFK1 treatment on the downregulation of eEF2 and CK18 (Figure 7C), suggesting that its downregulation may have contributed to the synergistic effects of the combined therapy.

## 3. Discussion

Tumor relapse and chemoresistance currently remain the major challenges in the treatment of HCC. The drugs used in current HCC treatment often target rapidly growing differentiated tumor cells but, in general, are not effective against CSCs. The ability to self-renew is essential for CSCs to expand their numbers and initiate tumor formation. In this study, we showed for the first time that rHGFK1 reduces the frequency of CD90+ CSCs in two HCC cell lines, ML-1 and HepG2, as indicated by the downregulation of the stemness-associated genes and the spherical formation ability of hyperspheres. HFGK1 also reduced the number of CD90+ CSCs in the ML-1-bearing orthotopic HCC mouse model. These results suggest that HGFK1 inhibits the self-renewal ability of CD90+ CSCs and promotes their differentiation in HCC.

CDDP is extensively used as a chemotherapeutic agent for the treatment of HCC, which usually causes chemoresistance and results in poor clinical efficacy [20]. Extensive evidence shows that pleiotropic alterations are frequently detected during the development of resistance to CDDP, which may be caused by the clonal evolution and expansion of CSCs [21]. In addition, although changes in a variety of genomics and epigenetic molecular elements are involved in the chemoresistance of CDDP, the alteration of stemness genes has not been reported. In this study, we showed that a low dose of CDDP (5 µg/mL) treatment significantly enhanced the percentage of the CD90+ sub-populations of CSCs in HepG2 cells and confirmed that this phenomenon was associated with the activation of the Wnt/β-catenin signaling pathway. This conclusion is consistent with reports that Dkk-1, an inhibitor of the Wnt signaling pathway, negatively regulated cellular resistance to cisplatin in brain tumors, head and neck cancer, and HCC [22]. Interestingly, we demonstrated that rHGFK1 treatment also reduced the frequency of CDDP-enriched CD90+ CSCs and Wnt/β-catenin signaling, suggesting that rHGFK1 may be a potential antagonist of Wnt signals in HCC.

As an evolutionarily conserved signaling module, Notch participates in embryonic cell-fate decisions and regulates stem/progenitor cell states. Notch1 and Notch2 are key regulators of liver development. Although the abnormal activation of the Notch pathway has been suggested to be involved in liver tumorigenesis, its role in the “stemness” properties of CSCs has not been clearly defined. The results from our study suggest that the notch pathway may play an important role in the maintenance of the self-renewal ability of CSCs and drug resistance. rHGFK1 treatment reduced the protein levels of Notch1 and Notch2, suggesting that Notch1 and Notch2 may be targets of rHGFK1. Thus, in this study, we revealed an even more broad spectrum of active mechanisms including Notch and Wnt/β-catenin. However, the possibility that the reduction in Notch could be due to the crosstalk between Notch and the Wnt/β-catenin pathway remains to be determined.

HGFK1 possesses a cluster including five residues that directly bind Met [8], the unique receptor of HGF. There is bidirectional regulation in that Met can activate β-catenin via AKT-dependent phosphorylation, while β-catenin/TCF transcriptionally activate Met [23]. Our study showed that rHGFK1 treatment reduced the protein level of pre-Met but not of mature Met, suggesting that Met is the direct target of rHGFK1, as the endocytosis of the ligand–receptor complex is expected to result in the downregulation of the receptor’s protein level.

Recently, Conley et al. showed that the anti-angiogenic agents sunitinib and bevacizumab increased the population of CSCs by generating intra-tumoral hypoxia in human breast cancer xenografts [24]. It has also been shown that the activity of the HGF/SF-Met pathway plays a critical role in hypoxia induced by anti-angiogenesis agents. As Endostatin has been shown to downregulate HIF-1α, we, therefore, propose that the synergistic effects of pHGFK1 and pEndo+pHGFK1 on CSCs in the tumor environment may be at least partly attributed to the combined effects of the receptor antagonistic function of HGFK1 on the HGF/SF-Met pathway and the inhibition of HIF-1α induced by the overexpression of Endostatin. These results suggest that the downregulation of pre-Met protein in rHGFK1-treated cells is attributed to the Wnt/β-catenin-mediated transcriptional inhibition of Met.

A targeted polymeric nanoparticle, formed by a biodegradable polymer and DNA, is one of the most desirable gene-therapy agents, as it can deliver therapeutic genes to the targeted organ and the tumor mass, thereby reducing side effects and enhancing drug efficacy [25]. In this study, we have successfully established an effective and long-lasting liver- and tumor-targeting gene delivery polymeric nanoparticle system, PH1 nanoparticles. PH1/pEGFP nanoparticles were absorbed through a superior mesenteric vein into the interstitial space via the leaking tumor microvessel and internalized by cancer cells, thus homing mainly to the tumor tissue of the liver. These results suggest that PH1 nanoparticles are effective delivery carriers for targeting the liver and the tumor.

Subsequent proteomic studies can help us to understand its molecular mechanism. Our results showed that pHGFK1 and pHGFK1+pEndo significantly reduce the expression of the epidermal growth-factor receptor pathway substrate 15 (Eps15) in vivo. Eps15 is a tyrosine-phosphorylated substrate of the EGFR kinase, which regulates EGF endocytosis [26]. Our previous study demonstrated the HGFK1 phosphorylation of EGFR in MEC cells [7]. These results indicated that HGFK1 may inactivate the EGFR and downregulate the expression of Eps15. Elongation factor 2 (EF2) is a critical enzyme governing the elongation of nascent proteins, which is upregulated in multiple cancers due to an adaptive response of cancer cells that resist the damaged micro-environments [27]. The downregulation of EF2 in the tumor tissue of mice treated with pHGFK1 and pEndo + pHGFK1 nanoparticles suggests that pHGFK1 and the combination of pEndo + pHGFK1 could inhibit the protein synthesis of cancer cells. Cytokeratin polypeptides 8 and 18 (CK8/CK18) are co-expressed as the obligate heteropolymers in adult hepatocytes, and the overexpression of CK8/CK18 was found in HCC with invasion properties [28]. Therefore, the downregulation of CK8/CK18 by pHGFK1 and pEndo + pHGFK1 nanoparticle treatments may contribute to reduced tumorigenesis and invasion.

## 4. Materials and Methods

### 4.1. Protein Expression and Purification

The template of an hHGF gene was isolated from the LO2 cells. An amplified cDNA fragment was subcloned into the *E*. *coli* expression vector pET24-a (Novagen, Madison, WI, USA) to produce pHGFK1. pHGFK1 was transformed into *E. coli* BL21(DE3), and the HGFK1 expression was induced by 1 mM IPTG. The cells were harvested via centrifugation for 30 min at 4000× *g*. The method of the expression and purification of the rHGFK1 polypeptides was previously described. Briefly, the cells were resuspended in 20 mM Tris–HCl, pH 8.0. The cells were incubated at 4 °C for 30 min; then, they were disrupted using a sonic homogenizer for 10 s, which was repeated six times with a 30 s interval between each time. After centrifugation at 4 °C, 12,000× *g* for 30 min, the pellet was collected and resuspended in 8 M urea, 0.1 M NaH_2_PO_4_, 10 mM Tris–HCl, pH 8.0. It was centrifuged again as before, and the supernatant was loaded on a Ni21-nitrilotriacetic acid-agarose column (Qiagen, Hilden, Germany). The recombinant protein was eluted from the column according to the manufacturer’s instructions. To achieve refolding, the purified protein was adjusted to pH 8.0, and DTT was added to a final concentration of 0.1 M. Following incubation at room temperature for 2 h, the solution was added to the refolding buffer at a ratio of 1:200 (*v*/*v*). After 24 h of incubation at room temperature, the renatured protein was dialyzed against distilled water for 24–48 h and lyophilized. The purity of the protein was detected via protein electrophoresis with Coomassie brilliant blue staining.

### 4.2. Cell Culture and MTT Assay

The mouse HCC ML-1 cell line was kindly provided by Dr. Che-Hsin Lee’s Laboratory (from Taiwan). Mouse endothelial CRL-2167 cells and HepG2 cells were purchased from ATCC. The attached cells were cultured in Dulbecco’s modified Eagle’s medium containing 10% fetal bovine serum, 1% glutamine, and 50 mg/μL gentamicin at 37 °C in 5% CO_2_. For the MTT assay, 3000 cells in 200 μL were added in triplicate to each well of 96-well tissue-culture plates and incubated at 37 °C (in 5% CO_2_). The cells adhered to the plate for about 8 h. The medium was replaced with 100 μL of fresh DMEM containing 2% FCS, including various dosages of rHGFK1, and rEndostatin and the combination of rHGFK1 and rEndostatin were added to each well, respectively. The medium of treatment was changed one time every day. After 72 h of incubation, 10 μL of MTT (100 μg/μL) were added to each well and incubated for another 4 h at 37 °C, 10% CO_2_. Then, 180 μL of medium were pipetted out from each well, 50 μL of DMSO were added, and the mixture was vortexed gently to dissolve the pellet. The absorbance at A_570_ nm, which correlates to the number of cells, was measured with a microplate reader (Model 450, Bio-Rad, Marnes-la-Coquette, France).

### 4.3. Flow-Cytometry Analysis and Single Cells Sorting

A total of 10,000 ML-1 cells were seeded in each of the six wells and cultured with DMEM including 2% BFA for 12 h before treatment. Next, 10 µg of rHGFK1 were put into a culture medium to treat the cells. The treatment medium was replaced once every 12 h. At 48 h after the treatment of rHGFK1, the cells were collected and stained with anti-mouse CD90-FITC (Biolegend, Cat:105305, San Diego, CA, USA) and anti-mouse CD133-FITC (Biolegend, Cat:141203, San Diego, CA, USA) antibodies overnight at 4 °C. Then, the cells were detected with a flow cytometer (BD LSRFortessa Cell Analyzer, San Jose, CA, USA), and the FACSDiva Version 6.1.3 software package was used to analyze the data.

The protocol of single-cell sorting followed the method described by Lee et al., with minimal adjustments [29]. Briefly, viable CD90+ cells from a single-cell suspension of ML-1 were sorted into a 15 mL tube containing capture medium (RPM1640 + 10%FBS + 1% antibiotics) by using a FACSAria cell sorter (BD, San Jose, CA, USA) equipped with an automated cell-deposition unit (ACDU) and by using a 488 nm laser light. For single-cell deposition, the cells were sorted by using a 100 μm nozzle with the sheath pressure set at 70 PSI using the sort precision mode set at the single cell. Dead cells were excluded from the sort based on their forward and side-scatter characteristics by using an electronic gate, before applying sort gates to define CD90+-expressing cells for collection. After sorting, the cells were washed with PBS and counted. The single cells were seeded with 2000 cells/mL in an ultra-low attached six-well plate and cultured with DMEM/F12 containing bFGF 20 ng/mL, EGF 20 ng/mL, insulin 10 µg/mL, and 2% B27 for three days. The morphology of the cells was monitored using a microscope, and sphere cells were used to isolate RNA for real-time PCR analysis.

### 4.4. Quantitative PCR (qPCR) Analysis

For the single-cell sorting experiment, the total RNA was isolated using Trizol reagent according to the manufacturer’s protocol (Invitrogen, Carlsbad, CA, USA). Glycogen (GlycoBlue™ Coprecipitant) was used to coprecipitate the RNA. Complementary DNA (cDNA) was synthesized by using a GeneAmp^®®^ Gold RNA PCR Kit (Applied Biosystems, Foster City, CA, USA) according to the manufacturer’s instructions. The amplification protocol consisted of incubations at 94 °C for 15 s, 63 °C for 30 s, and 72 °C for 60 s. The incorporation of the SYBR Green dye into the PCR products was monitored in real-time with an ABI 7900HT Sequence Detection System and with the SDS 1.9.1 software program (Applied Biosystems), and it was subsequently analyzed using the RQ Manager 1.2 software package (Applied Biosystems), thereby allowing the threshold cycle (CT) at which the exponential amplification of the products began to be determined. The amount of target cDNA was calculated relative to that of β-actin cDNA.

### 4.5. Polymer Synthesis and Plasmid Preparation

H1 and PH1 were synthesized by using a method described in our previous work [8,9]. For the synthesis of PEI-CyD-PEG, 100 mg of PEG (M.W. 3350, Sigma Company, Stockholm, Sweden) were dissolved in DMSO, activated using 10 mg of CDI (at room temperature for 3 h under nitrogen), dropped into 20 mL of DMSO containing 120 mg of PEI-CyD, and reacted for a further 24 h under nitrogen to form PEI-CyD-PEG. PEI-CyD-PEG was dialyzed for two days in DD water and freeze-dried. To form the bifunctional polymer (PH1), H1 and PEI-CyD-PEG were mixed with a ratio of nitrogen atoms to polymers of 1:1.

A CpG-free plasmid-encoding firefly luciferase (pORF-Luc) was purchased from In vivo Gene (San Diego, CA 92121, USA). The cDNA fragments containing IgK leader and the human Kringle 1 Domain of Hepatocyte Growth Factor (HGFK1) and human Endostatin (Endo) were subcloned into the pORF-Luc plasmid backbone with KasI and NheI to generate pORF-HGFK1(pHGFK1) and pORF-Endo(pEndo), respectively. The plasmids were transformed separately into competent DH5α cells, propagated in LB broth supplemented with 100 μg/mL of ampicillin, and purified with a PureLink^TM^ Hipure Plasmid Maxiprep kit (Invitrogen, Carlsbad, CA, USA). The quantity and quality of the purified plasmid DNA were assessed by measuring its optical density at 260 and 280 nm.

### 4.6. The Formation of H1/pORF-DNA and PH1/pORF-DNA Polyplexes

To prepare the H1/pORF-DNA and PH1/pORF-DNA polyplexes, the H1 and PH1 polymer solution was mixed with the plasmid DNA solution at an N/P ratio of 20:1 with an equal volume of 5% glucose. The polyplexes were incubated for 20 min at room temperature and filtered with 0.45 nm syringe filters before injection.

### 4.7. Cell Culture and Gene Transfection

The method of gene transfection with the H1, PEI-CyD, and PEI25KD polymers was described in our previous work [8,9]. Briefly, adherent cells were seeded at a density of 1 × 10^5^ cells/well in a 24-well plate the day before transfection for 24 h. An amount of 1 µg of pORF-EGFP or pORF-Luc in each well was mixed with polymers in a vortex and incubated for 15 min at room temperature to form polyplexes, respectively. The original cell culture medium was replaced with the complex solution containing the polyplexes and an additional 400 ul of OPTI-MEM for each well. After incubation for 4 h at 37 °C, the transfection medium was replaced with a fresh growth medium and further incubated for 24 or 48 h. To reveal the expression of enhanced green-fluorescence protein (EGFP) in the sample cells, a microscopic evaluation of EGFP expression was performed 48 h after transfection. To detect the expression of luciferase in the sample cell transfected by H1/pORF-Luc, a luciferase assay was performed 48 h after transfection. The detailed method is described below.

### 4.8. In Vivo Imaging

To determine the biodistribution of H1/pORF-Luc nanoparticles after their systemic administration, in vivo optical imaging of living Balb/c mice was performed using an IVIS 100 series imaging system (PerkinElmer, Waltham, MA, USA). Briefly, at the time point of detection, the mice were anesthetized, and D-luciferin (Xenogen) at a dose of 150 mg per gram of mouse body weight were injected intraperitoneally 5 min before the images were taken. The images were obtained at exposure times of 1 to 10 min, depending on the intensity of the emitted photons. The total photon flux (photons/second) in a region of interest (ROI) was quantitated using the Living Image^®^ Software package (version 4.3.1 PerkinElmer, Waltham, MA, USA).

### 4.9. Animal Model and Treatments

The animal experiment was conducted according to the guidelines set by the Animal Experimental Ethical Committee of Kunming Medical University, and the animal experiment was approved by the Animal Experimental Ethical Committee of Kunming Medical University (approval no. KMMU2021713, Approval date: February 2021). When ML-1 cells grew to an exponential growth phase, the cells were collected and used to establish an orthotropic mouse HCC model. Briefly, the Balb/c mice were anesthetized via intraperitoneal injection with a Hypnorm/Midazolam mixture (1:1:6); the dosage was 0.1 mL/10 gm. We removed the fur and made a 1 cm incision in the upper middle region of the abdomen. We exposed the left lobe of the liver and slowly injected a 10 ul cell solution (ML-1; 2 × 10^6^ cells) into the liver. The electro-coagulation probe was used to seal the puncture by briefly touching it with the tip of the needle before the needle was pulled out. We recovered the organ and closed the incision. Seven days after tumor-cell inoculation, the mice were randomly divided into four groups, which were treated via an intraperitoneal injection of PH1/pORF-HGFK1 (DNA 100 µg, N/P ratio: 20:1) polyplexes, PH1/pORF-Endo (DNA 100 µg, N/P ratio: 20:1) polyplexes, PH1/pORF-Luc (DNA 100 µg, N/P ratio: 20:1) polyplexes, and 5% glucose, respectively. Each group included six mice, and the experiments were repeated twice. In the first experiment, on the 30th day post-treatment, six mice of each group were sacrificed, the liver of each mouse was extracted, and photos were taken immediately. Then, the tumor masses were isolated and weighed. In the second experiment, eight mice from each group were treated with the mentioned reagents, and the survival of the tumor-bearing mice was monitored every day.

### 4.10. Immunohistochemistry

The paraffin-fixed tumor tissue was sectioned, and the standard avidin–biotin–peroxidase complex technique was applied for color development. The sections were dewaxed, soaked in ethanol, and then treated with 3% hydrogen peroxide to block endogenous peroxidase activity. Antigen retrieval was performed by microwave pretreatment. Non-specific immunoreactivity was blocked by incubating the sections in normal rabbit serum at room temperature and then incubating with primary monoclonal antibodies. To determine the microvessel density (MVD) and CD90-positive cells of tumorous tissue sections, anti-CD31 and anti-CD90 monoclonal antibodies (cell-signaling technology, dilution with 1:500–1:2000) were used to stain the tumor tissue, respectively. Afterward, the relative secondary biotinylated immunoglobulin was applied and then reacted with a streptavidin–biotinylated horseradish peroxidase complex. The sections were stained with a freshly prepared diaminobenzidine solution and then counterstained with Mayer’s hematoxylin. The negative control was obtained by substituting the primary antibodies with immunoglobulin G.

For the evaluation of MVD, the methods described in Gasparini’s criteria were used with minimal modification. Briefly, at a low power field (×40), the tissue sections were screened, and five areas with the most intense neovascularization (hot spots) were selected. Microvessel counts of these areas were performed at a high power field (×200). Any brown-stained endothelial cell or endothelial cell cluster that was separated from adjacent microvessels, tumor cells, and connective elements was counted as one microvessel, irrespective of the presence of a vessel lumen. The mean microvessel count of the five most vascular areas was taken as the MVD, which was expressed as the absolute number of microvessels per 0.74 mm^2^ (×200 field). For the evaluation of CD90-positive cells, any brown-stained cell was counted in one slide, five slides were randomly selected among the mice, and a total of three mice for each treatment were used for the calculation.

### 4.11. Two-Dimensional Electrophoresis (2-DE)

The method of two-dimensional electrophoresis (2-DE) was described in a previous study [30]. Briefly, 450 µg of protein from each sample was loaded and run in 24 cm-long pH 4–7 linear gradient ReadyStrip TM IPG Strips concurrently with 0.6% DTT and 1% immobilized pH gradient (IPG) buffer. Initially, 50 V was applied for 12 h to rehydrate each strip, following a voltage grade until 70 kWh. Subsequently, the strips were immersed in equilibration buffer I (50 mM Tris pH 8.8, 30% glycerol, 2% SDS, 6 M urea, and 1% DTT) for 15 min at room temperature and in the same conditions with equilibration buffer II (50 mM Tris pH 8.8, 30% glycerol, 2% SDS, 6 M urea, and 2.5% iodoacetamide). For the second dimension, proteins were resolved on 13% SDS-PAGE gels of 24 × 20 cm using the Ettan DALTsix vertical system. The 2-DE gels were covered twice with a fixation solution (50% methanol and 10% acetic acid, for 30 min) and washed twice with distilled water for 15 min per wash. The gels were then incubated two-fold with water-diluted Pro-Q DPS (120 min). For the removal of gel-bound nonspecific Pro-Q DPS, gels were distained four times with distaining solution (20% acetonitrile pH 4.0, 50 mM sodium acetate) for 30 min and washed with distilled water (twice, 5 min per wash). For image analysis, spots detected in at least two of three biological replicates were included. Experimental isoelectric point (pI) values of protein spots were assessed from their 2-DE gel position relative to linear gradient pH 4–7 focused strips, while experimental Mr values were obtained with molecular mass markers from 15 to 200 kDa.

### 4.12. Statistical Analysis

All experiments were performed in independent biological triplicates, and the results of the replicates were consistent. A *t*-test analysis was used for comparison between the two groups. One-way ANOVAs were performed for comparisons between more than two groups (GraphPad Prism 7, San Diego, CA, USA). Details of the number of biological replicates are described in the figure legends and Methods. Error bars represent standard deviations (mean ± SD). A *p*-value of <0.05 means that there was a significant difference, and a *p*-value of <0.001 was considered extremely significant.

## 5. Conclusions

HGFK1 inhibits the self-renewal ability and CDDP-induced chemo-resistance of CD90+ CSCs via the in vitro inhibition of the Wnt/β-catenin and Notch pathways and, consecutively, the in vitro downregulation of HGF/Met expression in HCC. In addition, endostatin can promote HGFK1’s effects on CD90+ CSCs, both in vitro and in vivo. A liver tumor-targeting, biodegradable, and effective polymeric nanoparticle system carrying pCpG-free plasmids has been successfully developed and validated in this study. The systemic administration of pEndo and pHGFK1 and the combination of pEndo with pHGFK1 nanoparticles all showed effective anti-tumor functions in an orthotropic HCC mouse model. Among them, the combination of pEndo with pHGFK1 showed the best anti-tumor efficacy via the inhibition of both neo-angiogenesis and CSCs of HCC. Their clinical application potential warrants further study.

## Data Availability

All data generated or analyzed during this study are included in this article and additional data are available from the corresponding author upon reasonable request.
